# Foam Splint—The Comfortable Way of Postoperative Immobilization After Surgical Hip Reconstruction in Children—A Randomized Clinical Trial

**DOI:** 10.3390/jcm14103485

**Published:** 2025-05-16

**Authors:** Manuel Gahleitner, Christina Haas, Gerhard Großbötzl, Matthias Christoph Michael Klotz, Tobias Gotterbarm, Lorenz Pisecky

**Affiliations:** 1 Department of Orthopedics and Traumatology, Johannes Kepler University Linz, Kepler University Hospital GmbH, 4020 Linz, Austria; manuel.gahleitner@kepleruniklinikum.at (M.G.);; 2 Department for Orthopaedics and Traumatology, Marienkrankenhaus Soest GmbH, 59494 Soest, Germany

**Keywords:** dysplasia of the hip, hip reconstruction, spica cast, foam splint, quality of life

## Abstract

Hip joint reconstruction is often necessary for children and adolescents with conditions like developmental dysplasia of the hip (DDH), neurogenic dislocation of the hip (NDH), or Legg–Calvé–Perthes disease (LCPD) when non-surgical treatments are ineffective. **Background:** Post-operative immobilization after hip reconstruction in children is crucial to promote proper healing and reduce the risk of complications. While spica casting has been the traditional method, it can lead to various issues. Foam splinting has emerged as an alternative approach. This study aimed to compare the effectiveness and satisfaction of the patient and the caregivers of spica casting and foam splinting after pelvic osteotomies in young patients with DDH, NDH, and LCPD. **Methods:** A prospective randomized clinical trial included patients aged 3 to 16 undergoing pelvic reconstruction (iliac and proximal femoral osteotomy, open reduction, and soft tissue procedures). Participants were randomized into two groups: one receiving spica casts and the other foam splints, both for a six-week period post-surgery. Quality of life (QOL) assessments like CPCHILD, SF-36, and EQ-5D were conducted using various scores to measure patient and caregiver satisfaction preoperative and at six and twelve weeks postoperative. The surgical techniques were consistent across both groups. **Results**: The study included 34 patients, with one excluded due to non-adherence. The spica cast group experienced statistically significant declines in QOL scores, while the foam splint group showed decreases that were not statistically significant. Complications were reported in 11 patients, with a higher prevalence in the spica cast group. **Conclusions**: The foam splint group demonstrated superior satisfaction levels and fewer complications, which leads to the conclusion that foam splinting should be the preferred option to spica casting for post-operative immobilization in these cases.

## 1. Introduction

Congenital disorders of the pelvic joint include developmental dysplasia of the hip joint (DDH) and deformities of the proximal femur.

Moreover, individuals with neuromuscular disorders often exhibit severe forms of neuromuscular dysplasia of the hip (NDH), resulting in luxation [1]. If conservative treatments prove unsuccessful, surgical intervention becomes necessary for reduction and reconstruction of the hip. There are no guidelines provided for the postoperative care of hip reconstruction in children in the current literature [2].

Outcomes can include pain during activities such as walking, standing, or sitting, and difficulties with walking, potentially leading to the loss of gait. Early detected cases of DDH or hip dislocation may initially be addressed through casting or splinting. Children with neuromuscular disorders more often experience luxation of the hip and unsuccessful conservative treatment [3]. In individuals with CP, various authors have demonstrated an occurrence of hip dislocation ranging from 18% to 60% in their reported patient populations [4].

In addition to DDH and NDH, pelvic reconstruction proves advantageous for young patients with Legg–Calvé–Perthes disease (LCPD) concerning the objective of hip containment, preventing further lateralization and supporting the remodelling of the femoral head [5,6,7,8,9]. Particularly in older children who have already developed the ability to walk, the presence of tightened soft tissue adds complexity to the procedure. In the majority of cases, a combination of interventions targeting both soft tissue and bone becomes essential to obtain joint reduction [8,9].

Following the surgical procedure, the traditional practice involves immobilization through spica casting for a six-week period, followed by subsequent physiotherapeutically controlled remobilization [10]. Many surgeons opt for casting to prevent re-dislocation, mostly applied in individuals with spasticity. Recognized complications include hygiene issues, skin lesions, neurological complications, and joint stiffness after casting. Some institutions undertake a spica cast change in a brief narcosis two weeks post-surgery. Another option for postoperative immobilization is foam splinting. This not only prevents the operated hip from secondary dislocation but also allows easy check-ups of the wound and facilitates physiotherapeutic manipulation of the ankle and knee, as well as the contralateral hip. Recent studies have demonstrated the safe application of foam splinting in terms of bone healing and a promise of fewer complications. In a retrospective study conducted by the group of Gather in 2018, it was shown that in relation to the postoperative X-ray a foam splint is comparable to a spica cast, with no significant differences in adverse events such as displacement of the bone transplant, necrosis of the iliac bone or femur, pseudarthrosis, or neurological complications [11]. Other colleagues, like Amen et al., tried to start an early functional mobilization for patients with cerebral palsy undergoing hip reconstruction without statistically significant results, considering revisions or complications [12].

Despite the available data on various possibilities of post-operative immobilization or care, no commonly accepted agreement on the optimal approach to postoperative immobilization in the treatment of surgically reconstructed pelvic joints has been achieved. In a randomized controlled clinical trial, the research team aims to demonstrate that the use of a foam splint results in a higher level of satisfaction among patients and their caregivers with the postoperative situation. Additionally, a reduced incidence of complications is anticipated. Potential advantages for patients include fewer complications and an improved quality of life throughout the aftercare period. Additionally, they do not require a second anesthesia for recasting. The primary hypothesis under investigation was whether the use of foam splinting for immobilization following hip reconstructive surgery results in greater patient satisfaction and higher quality of care compared to spica casting. This was assessed using the “Caregiver Priorities and Child Health Index of Life with Disabilities” as the measuring parameter (CPCHILD) [13,14]. Alternative issues of interest were adverse events, such as challenges with hygienic issues, sores, and neurological abnormalities. The primary endpoint of the trial was the participation in the 12-week post-interventional check-up and full completion of the questionnaire. As of now, no comparable study addressing this specific aspect has been conducted.

## 2. Materials and Methods

The research team at an Orthopedic Surgery department in Central Europe has formulated a unblinded, prospective randomized clinical trial to substantiate the main hypothesis: the use of foam splinting results in increased postinterventional level of satisfaction among the pediatric patients and their caregivers undergoing hip joint reconstruction. The study has been registered with the German Clinical Trials Register (DRKS-ID: DRKS00016861).

Recruitment was performed at the Clinic for Pediatric Orthopedic and Neuro-Orthopedic Surgery at the University Clinic by an experienced orthopedic surgeon. The eligible participants for inclusion were patients aged 3 to 16 years diagnosed with “congenital dysplasia of the hip”, “neuromuscular dysplasia of the hip”, and “Legg-Calvé-Perthes disease”, in need of pelvic reconstructive surgery. Exclusion criteria were a missing informed consent and non-cooperation.

The surgical criteria for inclusion comprised a Reimers migration index exceeding 40%, or 25–40% with observed progression, Tönnis hip classification II or worse, or an AC-Index exceeding the age-standardized Tönnis-classification.

Techniques used were the varisation derotation osteotomy of the femur (DVO), incomplete iliac osteotomy of Pemberton, Salter, and Chiari osteotomy, and Tönnis triple osteotomy in one specific case. Non-osseous techniques involved tendinous release of the psoas muscle, myofasciotomy of the hip adductors, knee flexors, and lengthening of the quadriceps tendon.

Participation required the completion of a standardized questionnaire before surgery, at 6 and 12 weeks postoperatively. Inclusion also mandated post-trial care during a standardized yearly routine check-up. All scheduled post-interventional check-ups were performed at the outpatient Clinic for Pediatric Orthopedic and Neuro-Orthopedic Surgery at the study site. Mandatorily, informed consent of the patient and legal guardian was collected by the surgeon prior to surgery.

**Figure 1:**
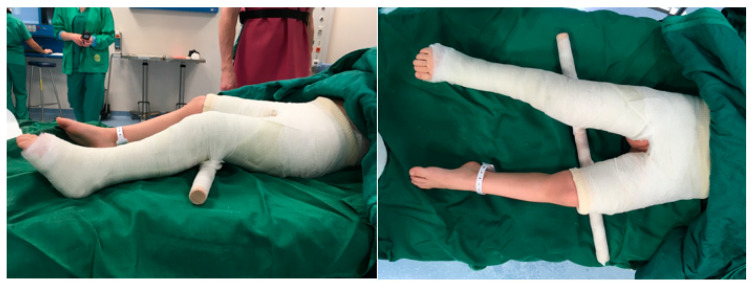
Post-operative spica cast with 10° flexion in the knee and 15° abduction, and 10° flexion in the hip of the affected leg. The non-affected leg is only treated with a shorter cast up to the knee [15].

Group A underwent treatment with a spica cast positioned in a slight hip flexion of approximately 10–15 degrees, with 10 degrees inward rotation and 30 degrees of hip abduction (Figure 1). A second brief general anesthesia, conducted two weeks post-surgery, was required for the removal of skin sutures and cast replacement. 

**Figure 2:**
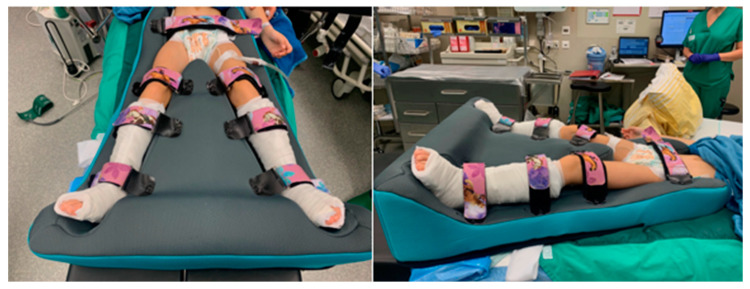
Post-operative foam splint including lower leg cast after additional procedure at the Achilles tendon.

Group B underwent treatment involving foam splinting for six weeks, positioning the hip in slight flexion (approximately 10–15 degrees), 10 degrees inward rotation, and 30 degrees of hip abduction (Figure 2). Notably, there was no requirement for a second anesthesia in group B. The cover of the splint is washable, and better physical therapy is possible for the non-affected leg. Additionally, lying 24/7 is much more comfortable, and the wound can be inspected at any time.

Throughout the immobilization period in this group, patient-specific physiotherapy for the lower extremity was administered based on the individual needs of the patient. The assignment process was random, using a computer-generated coin toss. The surgical technique was consistent across both groups, with no variations. To assess the quality of life and aftertreatment, standardized questionnaires were employed at the 6- and 12-week post-surgery intervals (refer to Table 1).

The utilized questionnaires included the “Caregiver Priorities and Child Health Index of Life with Disabilities” (CPCHILD), the “Short Form 36” (SF-36), and the “Euro Quality of Life 5D” (EQ-5D) [15,16,17]. Patient classification according to the “Gross Motorfunction Classification System” (GMFCS) scale was performed [18]. Exclusion criteria were a missing informed consent and rejection by the parents. Full data management was conducted pseudonymised. Blinding for outcome assessors was not considered because it did not seem feasible for us.

**Figure 3:**
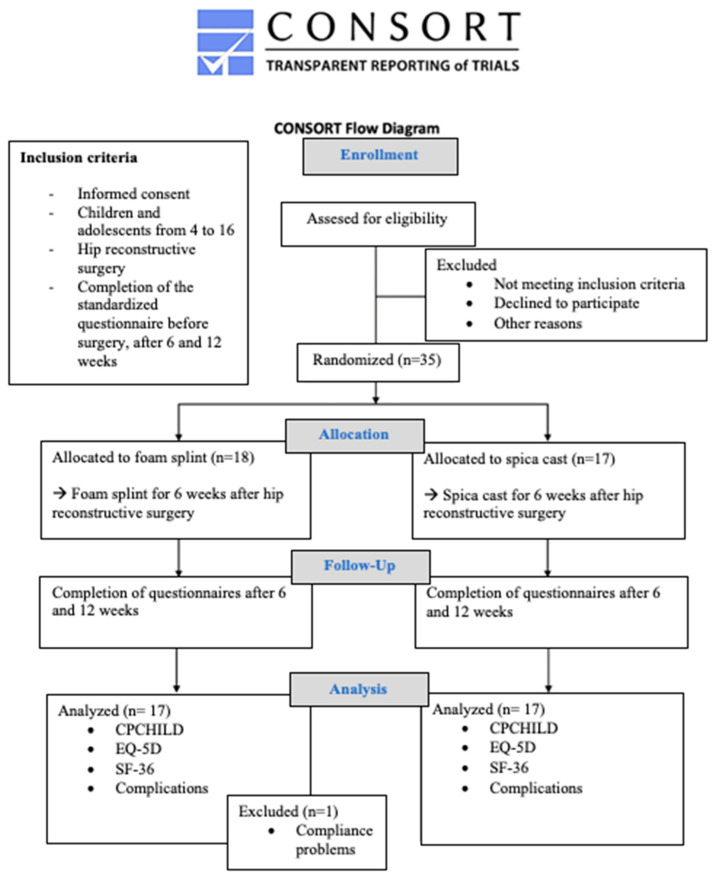
The algorithm of the trial with complete data; CONSORT diagram.

The statistical methods employed encompassed a comprehensive description of the epidemiological data, providing mean, standard deviation, minimum, maximum, and median for continuous data and scores, along with relative frequency for given variables. Main group characteristics were subjected to analysis using either a *t*-test or a Wilcoxon rank test. The reported *p*-values are indicated and considered statistically significant with values < 0.05. The calculation of sample size used a two-sided *t*-test, effect of Cohen d 1.1, alpha = 0.05, and 1-beta = 0.80, and resulted in a minimum of 15 patients per group. The process of enrollment is depicted in Figure 3.

## 3. Results

A total of 35 patients were allocated (Table 2) according to the study protocol. Due to severe compliance issues using the splint, a single patient needed to be excluded from the trial and the following analysis. This individual needed to be casted two weeks after surgery because of exacerbated epileptic seizures due to general anesthesia, further treatment was conducted without any complications. No additional data for this patient are given within the analysis. The full dataset for analysis was available for 34 patients, where 17 were treated with a spica cast and 17 with a foam splint.

Out of 23 patients with NDH, 1 was GMFCS type I, 2 type II, 3 type III, 2 type IV, and 15 type V. Of these cases, 13 received casting through the randomization process. The distribution regarding the GMFCS classification is detailed in Table 3. No underlying neurological condition like cerebral palsy could be identified in the remaining participants.

In 11 cases, complications occurred. Six in group A were treated with a spica cast, and five in group B were treated with a foam splint (Table 4). In the cohort using the spica cast, the material of one patient was already heavily soiled after one week. To prevent wound infection, the cast was changed. A pressure ulcer developed on a patient’s heel, which was discovered during a check after two weeks. The cast was shortened during the change to spare the heel, and the wound was locally treated. Regular dressing changes were performed in the outpatient clinic. A stage two decubitus ulcer in a child was successfully healed through local therapy. In another patient, a stage two ulcer developed after three weeks in the cast. In a challenging hygiene situation, one child developed an infection in the groin region five days postoperatively after an adductor tenotomy, requiring surgical wound revision. With antibiotic therapy, the infection healed within two weeks. After reconstruction and cast removal, there was a recurrence of femoral head lateralization in one patient. Regular follow-up appointments were temporarily arranged.

In the cohort using the foam positioning splint, two patients experienced minor sores; one was due to an adductor spasm, and the second was due to a slightly too tight-fitting splint. The splint was re-adjusted, and both children’s wounds healed rapidly with local therapy, regular dressing changes, and good padding.

In another patient, a deep wound infection occurred on the fourth postoperative day at the proximal lateral femur. The wound required surgical revision and antibiotic therapy, leading to healing within two weeks.

In one patient, a secondary suture had to be performed due to a ten-day wound dehiscence. In one case, knee flexor spasticity had to be treated with botulinum toxin and physiotherapy. The osteosynthetic material could be retained in all cases.

At the midterm follow-up, six weeks after initial surgery, all quality-of-life (QOL) scores revealed a decrease compared to the pre-interventional scores. Twelve weeks after intervention, the scores approximated the values obtained preoperatively (Table 5).

The equivalent numbers for group B (foam splint) also presented a decrease, notably not reaching statistical significance (Table 6).

**Figure 4:**
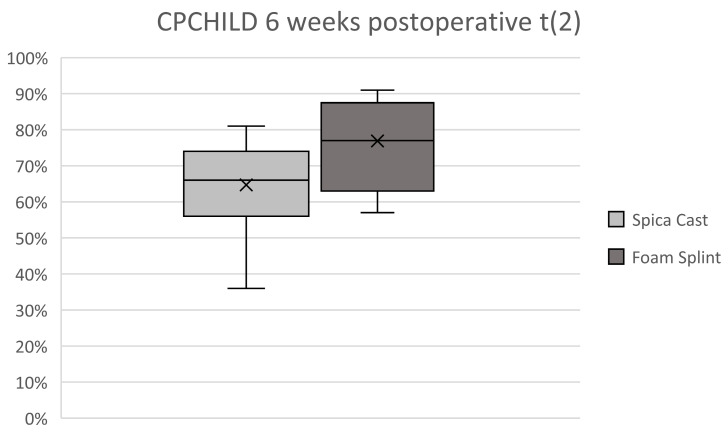
CPCHILD score 6 weeks post-surgery (t_2_).

As main findings supporting the target hypothesis, numbers for CPCHILD (Figure 4), EQ-5D, and SF-36 showed worse results in casting at the 6-week follow-up than in the foam splint group, and the difference reached statistical significance (CPCHILD 77%, 71–83 vs. 65%, 59–70, and *p* = 0.023). The 6-week difference diminished until the second follow-up after 12 weeks for all three QOL-scores, giving statistical significance for just SF-36 (SF36 90%, 75–100 vs. 84%, 75–100, and *p* = 0.017).

In our cohort, no participants experienced delayed bone healing or dislocation. After the six-week immobilization period, the postoperative rehabilitation protocol was applied equally in both groups. After six weeks of immobilization, either the cast or brace was removed, and participants were gradually mobilized with the help of physiotherapy. For patients without walking impairments, partial weight-bearing of the affected limb was prescribed, with full weight-bearing recommended only after 12 weeks postoperatively. For participants with walking impairments, transfer steps were allowed from the end of the immobilization period.

## 4. Discussion

The findings indicate beneficial data for the foam splint group compared to the plaster cohort concerning patient and caretaker satisfaction, and lead to our recommendation that post-operative care and immobilization should be performed using a foam splint if possible.

Of course, there is a discussion about whether a complex procedure such as hip joint reconstruction is necessary at all when there is the option of addressing the affected joint with a total hip arthroplasty (THA), thus avoiding extensive postoperative immobilization. Ref. [19] Our patient population is very young, which is why the decision is made early to pursue reconstruction. THA can only be implanted once the growth plate is fully closed, which usually occurs around the age of 15 years. In neurological patients, biological age, and therefore bone growth, often lags behind chronological age. This can lead to symptoms like pain, loss of gait or a shortened leg that cannot be resolved by either reconstruction if the femoral head is damaged or THA until the desired age for THA is reached. Furthermore, muscle shortening due to high hip dislocation in childhood poses a significant challenge for the implantation of THA. Our treatment plan is early reconstruction, followed by THA at an older age, if necessary, once normal muscle balance has been achieved.

The literature shows evidence indicating that prophylactic pelvic joint reconstruction has a positive impact on the personal wellbeing of children with cerebral palsy. In 2016, Di Fazio et al. demonstrated good effects of prophylactic surgery on the livability of immature with cerebral palsy [14].

Knowing the ongoing and long-lasting discussion of the ideal way of pelvic reconstruction, astonishingly, no consensus regarding the method or length of post-operative treatment has been reached [10].

Complications linked to spica cast immobilization are well-documented, with the recent literature reporting complications in the range of 4.5% to 13.4% in cases of hip reconstructive surgery and up to 28% in cases of proximal femoral fractures immobilized with a spica cast [20,21].

Pisecky et al.’s study group reported adverse events in 27.3 percent of the cases associated with casting in pelvic surgery. Adverse events were more prevalent in patients with neurological disorders (NDH) at 35% compared to those with developmental dysplasia of the hip (DDH) at 22%. Among the 23 adverse events reported, nine seemed to be associated with the spica cast immobilization [22]. 

Given data is mostly based upon narrative case series, and elaborate prospective randomized clinical trials were still missing.

Murgai et al.’s study group provided data on the use of foam padding in spica casts among 920 patients undergoing 2481 immobilizations. The study demonstrated decreasing numbers of adverse events for A-frame casts from 13.4% to 4.5%. Notably, individuals with neurological disorders exhibited fewer complications with a rate of 0.7%, and no cases of neurovascular deficits were reported in instances with foam padding, as opposed to a 4.5% occurrence in patients lacking foam padding [21].

The group of DiFazio reported adverse events in 28% of cases immobilized with casting for femoral fractures. The readmission rate was 31%, requiring recasting [20]. In a following prospective trial, the same group demonstrated a decrease of adverse events from 13.6 to 6.6 cases per 1000 castings by incorporating foam pads to improve the cast [23].

The study group of Gather implemented a simple protocol for early mobilization that included physiotherapeutic remobilization, allowing full weight-load four weeks after postoperative immobilization in a foam splint. The study results revealed no device-associated complications. Hence, it can be inferred that splinting is non-inferior to spica casting when it comes to postoperative immobilization for securing the osseous outcome [11].

Similarly, Vasconcellos et al. conducted a retrospective comparison of the clinical outcomes of two groups, where the first group was treated with a Spica cast and the second group with a foam pillow and stripes. The patient population was uniformly selected with cerebral palsy as the underlying condition, thus involving cases of neurological hip dysplasia or dislocations. In the clinical results, no statistically significant disadvantage in terms of bone healing during postoperative immobilization using the foam pillow was identified. Skin irritations were observed in both the cast group and the comparison group using foam pillows. Overall, the study concluded that the immobilization method should be chosen based on patient compliance [24].

Tabaie et al. retrospectively compared three different postoperative positioning methods in a uniform patient population with cerebral palsy following hip reconstruction. The patients were divided into three groups, and postoperative immobilization was carried out using either a Spica cast, a Petrie cast, or an abduction pillow. Again, no significant disadvantages were found among the three immobilization methods [25].

A wide range of post-interventional procedures exists and, in many clinics, the choice between them is often influenced by the surgeon’s expertise, personal preference, or longstanding tradition. This decision is considered a compromise, aiming to safeguard the results of the surgical intervention while preventing potential issues like superficial skin lesions or more severe complications associated with casting.

Surgeons often find themselves in a conflict of interest between preserving the achieved correction and avoiding complications such as skin problems. The fear of losing correction contrasts with the desire for a brief and uncomplicated period of immobilization, as preferred by both patients and caretakers. Frequently, minor adverse events like dermal sores, prolonged wound healing, and hygienic issues lead to unscheduled outpatient visits. More serious complications, such as deeper skin lesions, may necessitate unplanned hospital readmissions and, in some instances, surgical revisions.

To uphold patient autonomy and caretaker independence, efforts should be made to minimize readmissions and surgical interventions by addressing and preventing the underlying complications.

In an effort to enhance the evidence base for aftertreatment, this marks the inaugural prospective randomized clinical trial comparing casting and splinting for postoperative treatment in patients undergoing hip reconstructive surgery for neuromuscular dysplasia of the hip (NDH), developmental dysplasia of the hip (DDH), and Legg–Calvé–Perthes disease (LCPD). The primary objective of this study is to assess the quality of life and complication rates associated with these two distinct immobilization devices, and the data suggest encouraging outcomes.

Currently, spica cast immobilization remains a widely employed post-treatment protocol following hip reconstructive surgery in immature patients. The goal is to establish comprehensive criteria favoring the utilization of a foam splint over casting in the postoperative immobilization process after osseous hip surgery. There are certainly some challenges regarding the use and organization of a foam splint. It is clear that the splint can only be used for planned surgeries, as lead time is required for its production. Naturally, this type of treatment would also be desirable for proximal femur fractures in children, but this is not feasible.

In terms of cost, the production of the foam splint is certainly more expensive than a spica cast. However, overall, it is likely more resource-efficient for the local healthcare system. The need for a second general anesthesia for cast replacement is eliminated, along with a second hospital admission, and an additional slot in the theatre can be saved. 

Due to the randomization, no consideration was given to the underlying condition. However, postoperative handling proved to be particularly challenging in patients with severe epileptic seizures or limited communication and cooperation due to advanced infantile cerebral palsy. While the statistically significant results demonstrate clear benefits, especially for caregivers, from a medical perspective, cast therapy should be recommended in such cases with already known epileptic exacerbations. In patients whose seizure patterns change due to general anesthesia, it may be necessary to switch to cast therapy during the six-week immobilization period. However, delayed bone healing or an increased rate of pseudoarthrosis could not be documented even in the aforementioned patient group.

We think that the choice of postoperative immobilization has no impact on the long-term outcome. In our cohort, we did not observe any differences one year after surgery. In our opinion, the advantages of the foam splint are limited to the six-week immobilization period. During this time, it allows for better physiotherapy for the unaffected leg, enables wound inspection at any time, eliminates the need for a second general anesthesia for recasting, reduces pressure sores, and makes hygiene care significantly easier for caregivers.

We know that the sample size of only 34 participants is very limited; however, it was determined based on the power analysis and the ethical committee. The statistical values narrowly reach significance but are, in our opinion, still meaningful and reveal a significant decrease in the quality of life from the preoperative situation to the postoperative follow-up (t1) in the spica cohort. Conversely, the decline observed in the foam splint cohort during the same period does not reach statistical significance. At six weeks postoperatively, the data show favorable results for the foam splint compared to casting and support the main hypothesis of a beneficial immobilization technique for the patients and their caregivers.

### Strengths and Limitations

As the pioneer prospective randomized clinical trial exploring the use of foam splints and casts in postoperative immobilization following osseous pelvic surgery, the gathered data stands poised to contribute valuable insights to the decision-making process in postoperative care. This information is particularly pertinent to considerations of safety and wellbeing among both patients and caregivers. The planning of this clinical trial adhered to rigorous ethical and legal standards, receiving approval from ethical review committees. One limitation of the study is the heterogenous patient group (DDH, NDH, LCPD). This contributes to the fact that hip reconstruction is not the classic high-volume surgery in pediatric orthopedics. This limitation had to be accepted when planning and conducting the randomized trial in favor of timely data collection. A potential bias could also be the lack of blinding and the relatively small sample size. To further support these findings, larger studies with multicenter participation should be conducted.

## 5. Conclusions

The results indicate beneficial data for the foam splint group compared to the plaster cohort. Furthermore, detailed analysis highlights no statistically significant difference in the first postoperative follow-up (t2), confirming notably higher patient satisfaction in the foam splint cohort. In contrast, the changes in the group with a spica cast were statistically significant. This indicates that patients and their caregivers’ satisfaction significantly decreased in this group. As a result, foam splinting will be the standard mode of postoperative immobilization at the study site, whereas casting remains a backup method in certain selected cases, for example, in patients with frequent epileptic seizures or worse compliance, because due to clinical experience we now know, that the post-surgery handling is not that easy.

## Figures and Tables

**Table 1:** Schedule of enrollment, interventions, and assessments; t1—surgery; t2—6 weeks post-op; t3—12 weeks post-op [15].

	STUDY PERIOD				
	Enrollment	Allocation	Post-Allocation		Close-Out
TIMEPOINT	***−t*_1_**	**0**	***t*_1_**	***t*_2_**	***t*_3_**
ENROLLMENT:	X				
Eligibility screen	X				
Informed consent	X				
Allocation		X			
INTERVENTIONS:					
*[Spica Cast]*			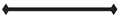		
*[Foam Splint]*			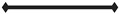		
ASSESSMENTS:		X	X	X	X
*[CPCHILD]*		X		X	X
*[SF-36]*		X		X	X
*[EQ-5D]*		X		X	X
*[Complications screen]*			X	X	X

**Table 2:** Epidemiologic data and procedures. (DDH—developmental dysplasia of the hip; NDH—neurological dysplasia of the hip; LCPD—Legg–Calvé–Perthes disease; SD—standard deviation; y—year).

	DDH	NDH	LCPD
N (hips)	6	23	5
age at surgery	5.96y; 3.15–10.81; SD 2.9	9.21y; 4.2–16.26; SD 3.3	6.61y; 61–7.62; SD 0.6
m:f	2:4	12:11	4:1
right:left	3:2	5:13	1:4
bilateral	1	5	0
Surgical procedure in detail			
Femoral osteotomy	5	23	5
Osteotomy of ilium	0	1	5
Salter osteotomy	0	0	5
Chiari osteotomy	0	1	0
Pemberton osteotomy	5	20	0
Psoas tenotomy	0	4	0
Adductor tenotomy	0	9	1
Open reduction	4	11	0
Hamstring lengthening	0	4	0
Lengthening of extension mechanism	0	1	0
Triple Osteotomy	1	0	

**Table 3:** Distribution regarding GMFCS classification.

NDH		
GMFCS	Foam Splint	Spica Cast
I	-	1
II	1	1
III	1	2
IV	1	1
V	7	8
	*n* = 10	*n* = 13

**Table 4:** Presenting all complications of both cohorts.

	Spica Cast	Foam Splint
Heavily soild	1	
Pressure ulcus	1	
Stage II decubitus	2	
Groin infection	1	
Postop lateralization	1	
Superficial skin lesion		2
Deep wound infection		1
Secondary suture		1
Botox injection due to knee flexor spasticity		1

**Table 5:** Comparison of the quality-of-life scores at two post-op assessments, 6 weeks and 12 weeks post-surgery (CI—confidence interval; SD—standard deviation).

	CPCHILD pre	CI	SD	EQ-5D pre	CI	SD	SF-36 pre	CI	SD
Total	79%	73–85	18	63%	55–71	24	83%	79–86	11
Foam splint	78%	68–88	21	62%	50–73	25	81%	76–86	11
Spica cast	81%	73–88	16	64%	53–76	24	85%	80–98	10
	CPCHILD t2	CI	SD	EQ-5D t2	CI	SD	SF-36 t2	CI	SD
Total	71%	36–89	13	50%	35–81	13	78%	66–87	6
Foam splint	77%	71–83	12	57%	50–64	15	81%	78–85	7
Spica cast	65%	59–70	12	43%	39–47	9	76%	73–78	5
diff	12%			14%			5%		
*p*	*p* = 0.023			*p* = 0.002			*p* = 0.004		
	CPCHILD t3	CI	SD	EQ-5D t3	CI	SD	SF-36 t3	CI	SD
Total	80%	75–85	16	68%	60–75	22	87%	84–89	9
Foam splint	84%	78–91	14	72%	61–83	23	90%	86–93	8
Spica cast	76%	69–84	16	63%	53–73	21	84%	80–87	8
diff	8%			11%			6%		
*p*	*p* = 0.093			*p* = 0.301			*p* = 0.017		

Comparing the QOL-scores for each group, a significant decrease was observed in group A (cast) pre- vs. post-interventional (t2) for the scores CPCHILD (81%, 73–88, SD 16 vs. 65%, 59–70, SD 12; *p* < 0.001), EQ-5d (64%, 53–76, SD 24 vs. 43%, 39–47, SD 9; *p* = 0.002) and SF-36 (85%, 80–98, SD 10 vs. 76%, 73–78, SD 5; *p* = 0.002).

**Table 6:** QOL scores pre- and post-op by a mode of immobilization (SD—standard deviation).

	CP	Low–High	SD	EQ-5D	Low–High	SD	SF-36	Low–High	SD
Spica cast pre	81%	73–88	16	64%	53–76	24	85%	80–98	10
Spica cast post1 (t2)	65%	59–70	12	43%	39–47	8	76%	73–78	5
diff	16%			21%			9%		
*p*	*p* < 0.001			*p* = 0.002			*p* = 0.002		
	CP	low–high	SD	EQ-5D	low–high	SD	SF-36	low–high	SD
Foam splint pre	78%	68–88	21	62%	50–73	25	81%	76–86	11
Foam splint post1 (t2)	77%	71–83	12	57%	50–64	15	81%	78–85	7
diff	1%			4%			0%		
*p*	*p* = 0.332			*p* = 0.136			*p* = 0.796		

## Data Availability

The data presented in this study are available on request from the corresponding author. The data are not publicly available due to data protection policy involving children and patronized subjects.

## Abbreviations

The following abbreviations are used in this manuscript:

DDH	Developmental dysplasia of the hip
NDH	Neurological dysplasia of the hip
LCPD	Legg–Calvé–Perthes disease
CP	Cerebral Palsy
CPCHILD	Caregiver Priorities and Child Health Index of Life with Disabilities
AC-Index	Acetabular Index
DVO	Derotation varisation osteotomy
SF-36	Short Form 36
EQ-5D	Euro Quality of Life 5D
SD	Standard Deviation
GMFCS	Gross Motorfunction Classification System
THA	Total Hip Arthroplasty
QOL	Quality of Life
